# Iontophoresis and electroporation-assisted microneedles: advancements and therapeutic potentials in transdermal drug delivery

**DOI:** 10.1007/s13346-024-01722-7

**Published:** 2024-10-21

**Authors:** Mehrnaz Abbasi, Braeden Heath

**Affiliations:** 1 College of Human Sciences, Department of Nutritional Sciences, Auburn University, Auburn, AL 36849 USA; 2 College of Sciences and Mathematics, Department of Biomedical Sciences, Auburn University, Auburn, AL 36849 USA

**Keywords:** Transdermal drug delivery, Microneedle, Iontophoresis, Electroporation, Therapy

## Abstract

Transdermal drug delivery (TDD) using electrically assisted microneedle (MN) systems has emerged as a promising alternative to traditional drug administration routes. This review explores recent advancements in this technology across various therapeutic applications. Integrating iontophoresis (IP) and electroporation (EP) with MN technology has shown significant potential in improving treatment outcomes for various conditions. Studies demonstrate their effectiveness in enhancing vaccine and DNA delivery, improving diabetes management, and increasing efficacy in dermatological applications. The technology has also exhibited promise in delivering nonsteroidal anti-inflammatory drugs (NSAIDs), treating multiple sclerosis, and advancing obesity and cancer therapy. These systems offer improved drug permeation, targeted delivery, and enhanced therapeutic effects. While challenges remain, including safety concerns and technological limitations, ongoing research focuses on optimizing these systems for broader clinical applications. The future of electrically assisted MN technologies in TDD appears promising, with potential advancements in personalized medicine, smart monitoring systems, and expanded therapeutic applications.

**Graphical Abstract:**

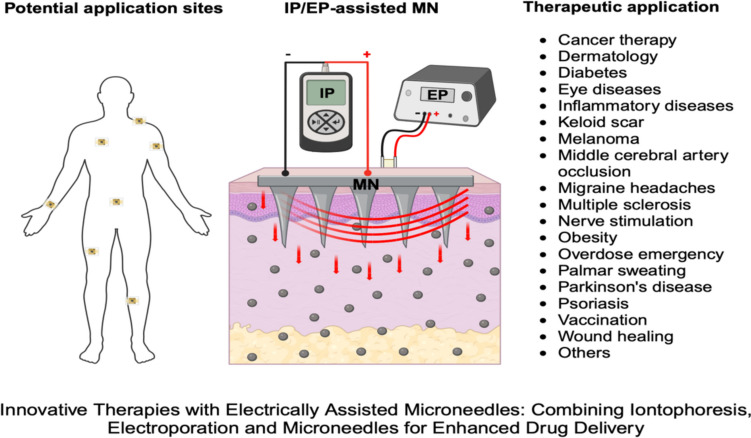

## Skin structure

The skin is the body's largest organ and is the main protective barrier against various external threats, such as harmful substances, allergens, and microorganisms. It helps maintain the body's internal balance by preventing excessive water loss and shielding against the damaging effects of ultraviolet radiation. Additionally, the skin contains specialized receptors that respond to environmental stimuli, including pressure, pain, and temperature changes. Altogether, these functions demonstrate the skin's crucial role in safeguarding overall health and facilitating interaction with the external world [1–4]. The skin comprises multiple layers of cells and tissues that connect to underlying structures through connective tissue. The primary layers of the skin are the epidermis, dermis, and subcutaneous tissue (Hypodermis) [2, 5]. The epidermis is the outermost layer divided into four sublayers: basale, spinosum, granulosum, and corneum. The corneum layer acts as a strong barrier against drug diffusion. The dermis beneath the epidermis is richly supplied with blood vessels and nerves, providing nutrients to the skin's cells and removing waste products. It also allows for the systemic absorption of drugs. The hypodermis is the deepest layer of the skin and is not always considered part of the skin. The skin appendages, including sweat and sebaceous glands, hair, and hair follicles, are associated with the dermis [4]. The thickness and composition of the skin layers, epidermis, and dermis vary throughout the body. Each skin layer has a specific shape, morphology, position, and state of differentiation of keratinocytes, which define the layer accordingly. Keratinocytes are the primary components of all skin layers except for the Stratum Basale. Variations between skin layers can occur due to differences in the differentiation state of keratin-producing cells [1, 6].

### Epidermis

The epidermis is a layer of skin that originates from the ectodermal tissue [7]. The epidermis comprises different types of cells, with keratinocytes and dendritic cells being the two main types. Other cell types, such as melanocytes, produce pigments and originate from the neural crest. There are also Langerhans cells, which are mobile antigen-presenting cells, and Merkel cells, which have epithelial and neuroendocrine characteristics. Melanin is the pigment that gives color to our skin, and melanocytes produce it. The transfer of melanin granules from melanocytes to neighboring epidermal cells involves long processes. These processes carry the melanin granules to the cytoplasm of basal keratinocytes, where the melanin is then transferred to neighboring keratinocytes through a process called "pigment donation." This process involves the phagocytosis of the tips of melanocyte processes by keratinocytes. Langerhans cells are dendritic cells and act as the first line defenders of the skin. They are important in antigen presentation and are primarily found in the stratum [2, 6–8]. The epidermis has five layers: the stratum basale, stratum spinosum, stratum granulosum, stratum lucidum, and stratum corneum (SC). Each layer of the skin has specific structural proteins that define it. In the stratum spinosum, the proteins keratins, involucrin, and transglutaminase-1 are expressed. On the other hand, the stratum granulosum expresses the late differentiation markers loricrin and filaggrin [9]. The stratum basale is the deepest layer separated from the dermis by the basement membrane. It contains cuboidal to columnar stem cells that constantly produce keratinocytes and melanocytes. The stratum spinosum, located above the stratum basale, has irregular, polyhedral cells with spines that extend outward and contact neighboring cells. Dendritic cells can also be found in this layer. The stratum granulosum, which is the third layer, contains diamond-shaped cells with keratohyalin and lamellar granules. The keratohyalin granules contain keratin precursors that eventually form bundles, and the lamellar granules contain glycolipids that function as glue to keep the cells stuck together. The fourth layer, the stratum lucidum, is only present in the palms and soles of thicker skin. It is a thin, clear layer consisting of eleidin, a transformation product of keratohyalin. Finally, the stratum corneum is the uppermost layer and comprises 20–30 cell layers of dead keratinocytes, known as anucleate squamous cells. This layer varies in thickness, especially in callused skin. The dead keratinocytes secrete defensins within this layer, part of our first immune defense [2, 8, 10]. The thickness of the epidermis varies depending on its location. For instance, it is around 40 µm on the eyelids and 1.6 mm on the palms and soles of the feet. Moreover, the thickness of the epidermis in other parts of the body is as follows: 12.7 µm on the dorsal part of the forearm, 13.5 µm on the shoulder, 16.1 µm on the buttock, and an average of 16.6 µm on all body sites. The skin thickness for the triceps is approximately 6 mm, while it is 15 mm on the anterior abdomen and 8 mm on the anterior thigh. The epidermis has a normal turnover rate of about 28 days [1, 2, 5, 6, 10–13].

### Stratum corneum (SC)

As skin cells differentiate and move towards the skin's surface, they undergo programmed destruction. These differentiated cells are called corneocytes, containing only keratin filaments embedded in a filaggrin matrix. Cornified lipid envelopes replace the plasma membranes of the previous keratinocytes, and the cells flatten, connecting with corneodesmosom and stacking as layers to form the SC [14]. SC is composed of a network of corneocytes and an extracellular lipid matrix. This layer acts as a two-compartment system, where the lipid-enriched matrix separates the hydrophobic, protein-rich corneocytes from the external environment. Ceramides, free fatty acids, and cholesterol are major lipid types in SC. The network is arranged in a "bricks and mortar" structure, with the extracellular matrix forming lamellar membranes [14, 15]. SC inhibits extra water loss from the body and, on the other hand, restrains the entrance of insoluble and high-molecular-weight topical medications. SC has about 18 to 21 layers. The SC thickness varies from 5 to 10 μm in the normal mammalian epidermis. Any defects in protein or lipid can lead to problems with the stratum corneum, causing abnormalities such as parakeratosis and scaling. These issues can result in skin diseases such as psoriasis, chronic eczema, and squamous cell carcinoma [16, 17].

### Dermis

The dermis, situated between the subcutaneous layer and the dermal–epidermal junction, is a crucial component of the skin, often referred to as the "true skin." As the thickest skin layer, it provides essential support to the epidermis. Composed primarily of fibrous, filamentous, and amorphous connective tissue, the dermis houses various structures and cell types, including nerve endings, blood vessels, epidermal appendages, fibroblasts, macrophages, and mast cells. Fibroblasts play a key role in the dermis by synthesizing and secreting collagen, the primary structural protein of this layer. The dermis contains blood-derived cells such as lymphocytes, plasma cells, and other leukocytes that enter the tissue in response to various stimuli. This layer is crucial in determining the skin's physical properties and contributes to its flexibility, elasticity, and tensile strength. These characteristics are essential for the skin to withstand mechanical stress and maintain its integrity [1, 10, 18]. The dermis is divided into two distinct regions: the papillary dermis and the reticular dermis. The papillary dermis, the uppermost layer, comprises loose areolar connective tissue containing collagen and reticular fibers. This layer is rich in fibroblasts, blood vessels, and a few adipocytes. It also houses various defensive cells, lymphatic capillaries, nerve fibers, and specialized touch receptors called Meissner corpuscles, contributing to skin nourishment and sensory perception. Beneath the papillary layer lies the thicker reticular dermis, primarily consisting of dense collagen bundles that anchor the skin to the underlying subcutaneous tissue. The reticular dermis contains abundant sensory and sympathetic nerves, sweat glands, and hair follicles [18, 19]. The dermis ranges from 2 to 5 mm thick and predominantly comprises collagenous fibers (70%) and elastin fibers. These components play crucial roles in providing skin strength and elasticity, respectively. The complex structure and composition of the dermis enable it to perform its various functions in supporting and nourishing the skin [20, 21].

### Hypodermis

The hypodermis, the subcutaneous layer or superficial fascia, is immediately beneath the dermis. This layer is a connective interface between the skin and the underlying bones and muscles, providing physical protection through its fibrous tissue composition. The hypodermis is characterized by its loose, areolar connective tissue and adipose tissue, which are well-vascularized. The hypodermis plays a crucial role in thermoregulation and maintaining skin stability by linking the dermis to internal organs. One of its primary functions is fat storage, facilitated by the presence of adipose tissue. Notably, the fat content of the hypodermis is not uniform; it varies depending on factors such as species, anatomical location, and the individual's nutritional status. This adaptable layer contributes significantly to the skin's overall function and appearance while also serving as an important energy reserve for the body [6, 22, 23]. Above the subcutaneous white adipose tissue (sWAT) lies a distinct layer rich in adipocytes, known as dermal WAT (dWAT). This specialized layer plays a multifaceted role in various skin-related processes. dWAT regulates hair follicle cycling, facilitates wound healing, and maintains temperature homeostasis. Additionally, it contributes to cutaneous fibrosis processes, provides protection against skin infections, and participates in other important skin phenomena. The diverse functions of dWAT highlight its significance in maintaining skin health and its involvement in various dermatological processes, distinguishing it from the deeper subcutaneous fat layer [24]. The hypodermis contains abundant fat-storing cells that serve as an energy reserve, provide insulation, and protect underlying structures. Fat accumulation and distribution are influenced by genetics, hormones (like insulin, glucagon, leptin, and estrogen), age, and gender. As people age, fat distribution changes. Gender-specific patterns emerge, with men typically accumulating fat in the neck, arms, lower back, and abdomen, while women tend to store fat in the breasts, hips, thighs, and buttocks. These factors contribute to individual variations in body composition and shape [25].

### Mechanisms of transdermal drug penetration

Drug penetration through intact skin occurs via two primary routes: trans-epidermal and trans-appendageal. The trans-epidermal pathway involves the SC and consists of two sub-routes: the intracellular route for hydrophilic or polar substances and the intercellular route for lipophilic or non-polar substances. These pathways allow for the selective transport of different molecules across the skin barrier, with the intracellular route favoring water-soluble compounds and the intercellular route accommodating fat-soluble substances [26]. The trans-appendageal route, involving hair follicles and sebaceous glands, plays a notable role in the penetration of steroids and potentially other molecules with similar properties [15, 27]. Despite comprising only about 0.1% of the total skin surface in humans, these appendageal pathways have gained renewed interest in recent years. While historically underestimated due to their limited surface area, contemporary research has highlighted the potential significance of hair follicles in facilitating drug permeation through the skin. This reevaluation has led to increased recognition of appendageal routes as promising avenues for enhancing transdermal drug delivery, challenging previous assumptions about their relative importance in skin penetration processes [28].

The use of the skin to deliver different kinds of compounds has been studied extensively for centuries [29]. Understanding the structure of the skin and the mechanisms of drug absorption has dramatically advanced the development of transdermal drug delivery systems (TDDS). As a result, researchers have developed methods to improve drug absorption through the skin by modifying the structure of the SC or altering drug penetration routes, either chemically, physically, or using a combination of these methods [26]. In the following sections, we will discuss the development of TDDS and various technologies for enhancing drug absorption through the skin.

## Transdermal drug delivery systems (TDDS)

Over the past decade, researchers have intensively explored effective methods for delivering drugs through the skin. This focus stems from the desire to develop innovative drug delivery systems that address the drawbacks associated with conventional administration routes, particularly injections. Injections often cause pain, trigger needle phobia, and carry the risk of transmitting infectious diseases, leading to reduced patient compliance. TDDS offers a painless alternative, where drug formulations are applied directly to healthy skin. In this approach, the SC serves as the initial barrier to penetration. Once past the SC, the drug must traverse the deeper layers of the epidermis and dermis to reach its target. This non-invasive method aims to enhance patient comfort and adherence while maintaining therapeutic efficacy, making it an attractive area of research in pharmaceutical sciences [26, 30–32]. It can deliver drugs at a controlled rate, providing constant and continuous administration and eliminating unwanted adverse effects [33].

TDDS offers numerous advantages over traditional oral or injectable routes. These systems enable the controlled release of medication, bypass first-pass metabolism, and avoid gastrointestinal degradation. They help maintain steady-state plasma concentrations and allow for individualized dosing options. The skin's barrier function is a rate-limiting step, which can benefit prolonged drug delivery with short half-lives. Enhanced delivery technologies can target local or systemic effects, reducing dosing errors and altering pharmacokinetic profiles [32, 33]. TDDS provides a steady, prolonged drug infusion, reducing adverse side effects and minimizing therapeutic failures associated with intermittent dosing. This method is particularly beneficial for patients who cannot tolerate oral medications due to issues like vomiting. By bypassing the gastrointestinal tract and liver, TDDS enhances therapeutic efficiency and avoids first-pass metabolism. This non-invasive approach improves patient compliance, allows easy self-administration, and enables immediate cessation of drug input by simply removing the patch. TDDS also reduces the need for multiple doses due to controlled drug release. Furthermore, the large surface area of the skin facilitates enhanced absorption of various drugs, making TDDS an attractive option for pharmaceutical delivery [26, 34–36]. These benefits collectively lead to more consistent drug levels, potentially lower overall doses, reduced dosing frequency, improved bioavailability, and better therapeutic outcomes. TDDS can provide precise, patient-specific dosing while minimizing side effects and dosing errors. However, the specific impact on dosing depends on the drug's properties, the patch design, and individual patient factors, requiring careful consideration in developing and prescribing these systems [26, 33, 37, 38].

Despite its advantages, TDDS faces several limitations. The skin's inherent impermeability restricts the range of drugs that can be effectively delivered through this route. Some patients may experience skin irritation or dermatitis at the application site, potentially leading to treatment discontinuation. TDDS is not suitable for drugs requiring high blood concentrations, and the cost of developing and manufacturing these systems can be prohibitive in some cases. While TDDS offers numerous benefits, these limitations must be carefully considered and addressed before it can fully replace oral delivery methods or hypodermic injections in many therapeutic applications. Ongoing research aims to overcome these challenges and expand the potential of TDDS in drug delivery [31, 32].

### Properties that influence TDD

The factors influencing TDD are complex and varied, involving physicochemical, biological, environmental, and physiological aspects [26, 39]. Physicochemical properties such as molecular weight, lipophilicity, melting point, and solubility play a crucial role in determining a drug's suitability for transdermal administration. Ideally, drugs should have a molecular weight below 500 Da, as larger molecules struggle to penetrate the SC [40–42]. Moderate lipophilicity, characterized by a log P value between 1 and 3, is preferred because it allows the drug to partition effectively into the skin's lipid and aqueous environments [43–46]. Lower melting points (below 200 °C) generally correlate with better skin permeation, while sufficient solubility in both lipophilic and hydrophilic environments is essential for crossing the SC and viable epidermis [46]. Half-life, a partition-coefficient (P) or distribution-coefficient (D) (which is defined as a concentration ratio of a solute in one phase or solvent compared to another), volume of distribution, total body clearance, therapeutic plasma concentrations, and bioavailability factors are other physicochemical properties which are also important for consideration [39, 47].

Biological factors also significantly influence drug absorption through the skin. Skin thickness varies across different body sites, impacting drug permeation rates. For instance, the skin on the eyelids is approximately 0.5 mm thick, while that on the forearm is about 1.3 mm, and the palms and soles can be as thick as 4 mm [48–51]. Age-related changes further complicate this landscape; infant skin is more permeable than adult skin due to its incomplete barrier function, while elderly skin exhibits reduced barrier integrity, decreased hydration, and altered lipid composition, all of which can affect drug permeation [52–55]. Body weight and fat distribution also play a role; obesity can alter drug pharmacokinetics due to increased adipose tissue, potentially impacting transdermal absorption and distribution. Gender differences in fat distribution may also influence absorption patterns [56–59]. Skin hydration is another critical factor, as well-hydrated skin generally allows for better drug permeation [60, 61]. Additionally, skin metabolism can affect drug bioavailability, as enzymes present in the skin may metabolize certain drugs before they can enter systemic circulation [62, 63]. Blood flow to the skin can enhance absorption, as increased cutaneous blood flow facilitates the delivery of drugs to the systemic circulation [50].

Environmental and physiological factors further influence TDD. Temperature is a significant factor; higher skin temperatures can increase drug permeation by enhancing diffusion and blood flow [64–66]. Sunlight exposure thins blood vessels and causes bruises on the sun-exposed skin site. It also can induce pigment changes. Cold weather results in the dry and itchy skin. Acne or skin spots on the face and surface result from bacteria increment and pores closer in the presence of air pollutants, which can change drug delivery across the skin. They also can interfere with the skin barrier and its natural protection system and affect skin moisture [65, 67, 68]. The pH of the skin surface, typically ranging from 4.5 to 6.5, can affect drug ionization and, consequently, its permeation [69, 70]. Furthermore, the application site is important, as different body areas exhibit varying permeability due to differences in skin thickness, hydration, and follicular density. Lastly, the condition of the skin itself plays a crucial role; diseased or damaged skin may exhibit altered permeability compared to healthy skin [50, 71, 72]. Understanding and optimizing these factors is crucial for developing effective TDDS tailored to specific drugs and patient populations, enhancing drug absorption, and improving therapeutic outcomes in transdermal applications.

### TDDS general classification

TDDS can be broadly classified into several categories based on their design and mechanism of action. The main types include matrix, reservoir, drug-in-adhesive, and micro-reservoir systems. Matrix systems contain the drug dispersed in a polymer matrix, while reservoir systems have a drug core surrounded by a rate-controlling membrane. Drug-in-adhesive systems incorporate the drug directly into the adhesive layer. Micro-reservoir systems combine elements of both matrix and reservoir designs [73, 74].

Advanced TDDS employs innovative techniques to enhance skin permeability and drug administration [34]. These include active systems using external energy sources (e.g., iontophoresis (IP) and electroporation (EP)) and passive systems utilizing chemical enhancers or nanocarriers [75]. Microneedle (MN) technology creates painless micro punctures for drug delivery [74]. These advanced methods enable the transdermal administration of a wider range of molecules, including large proteins and vaccines, offering tailored drug release profiles and improved skin permeation. By addressing the limitations of conventional systems, these innovations expand the possibilities for controlled and efficient transdermal drug delivery, potentially providing alternatives to traditional injections and oral medications [29, 43].

### Electrical techniques

Electrical enhancement techniques, such as IP and EP, offer precise control over drug release rates and have shown promise in improving the efficiency and effectiveness of TDDS, expanding the range of drugs that can be administered through the skin [29, 34].

#### *Iontophoresis (IP)*

Iontophoresis (IP) is a non-invasive drug delivery method that uses low-intensity electric current to help transport charged molecules across the skin. This technique usually involves currents from 0.1 to 1.0 mA/cm2 to create potential gradients that drive ionic drugs through the skin via electrostatic effects. By applying an electrical current, IP improves the permeation of charged therapeutic agents into and through the skin barrier, providing an alternative to traditional needle-based injections for drug administration. IP operates using carbon-fiber electrodes connected to iontophoretic barrels on a power supply, often referred to as a dose controller or power unit. This method is generally considered non-quantitative because accurately measuring the amount of drug delivered can be challenging. To estimate the drug quantity delivered via electrical application, one must correlate the drug's potency with various ejection currents. Commercially available iontophoretic devices include Phoresor®, Lidosite®, E-trans®, IONSYS®, Zecuity®, Microphor®, Dupel®, Macroduct®, Nanoduct®, and Drionic® which are used for local anesthesia, pain management, anti-inflammatory treatments, diagnostic procedures, and hyperhidrosis therapy [26, 32, 76, 77]. Successful iontophoretic drug delivery depends on several factors, such as the pH of the donor solution, electrode type, buffer concentration, and the molecular size of the drug [78–80]. The exact mechanism of IP is not fully understood, but it provides reliable and effective treatment when applied with proper technique and timing [81]. Iontophoretic transport involves two primary mechanisms: electromigration (also known as electrorepulsion) and electro-osmosis. Electromigration refers to the movement of ions towards the oppositely charged electrode. In contrast, electro-osmosis involves transporting water molecules from the anode (positive electrode) to the cathode (negative electrode) [82]. Several factors influence iontophoretic drug delivery, including drug concentration, total charge, physicochemical properties of the molecule, molecular size, partition coefficient, and the skin area used for application. While IP generally favors the transfer of small, hydrophilic molecules, research has shown that larger molecules, such as peptides, can also be delivered using this method [79, 80]. The effectiveness of iontophoretic transfer is also affected by skin surface integrity, thickness, and variations in blood flow across different skin areas [82, 83]. IP is generally associated with mild adverse effects rather than severe ones. When proper application techniques are used, the treatment is typically well-tolerated [81, 84].

The IP device consists of a power unit and two electrode compartments. The drug formulation, containing ionized molecules, is placed in the electrode compartment with the same charge, while the counter electrode is positioned at a distant site on the skin. The Ag/AgCl electrode pair is commonly used for IP. When current is applied, cations and anions migrate in opposite directions from their respective anode and cathode compartments [80]. The current applied in IP can be direct, alternating, or pulsed, taking various waveforms such as square, sinusoidal, triangular, or trapezoidal. These variations in the current type and waveform can significantly impact the efficacy of transdermal drug delivery [85]. IP application on the skin has several limitations. Patients may experience tingling or itching sensations, which depend on the applied current density. While uncomfortable, these sensations are generally not harmful. Skin irritation can occur at both the anode and cathode sites, and erythema is a potential side effect, with its severity varying based on factors such as dose, frequency, and duration of application. Material defects, particularly those arising from skin-metal contact, can cause skin injuries. Additionally, IP systems are generally more expensive than conventional topical formulations [80, 82].

#### *Electroporation (EP)*

EP is an electrical technique that temporarily disrupts cellular membranes, creating transient pores in the skin within milliseconds. This method involves applying approximately 100 V for 10 μs to 10 ms, which generates aqueous pores in the stratum corneum's lipid bilayers. While effective for enhancing transdermal drug delivery, EP can cause discomfort, including pain and muscle contractions. These side effects occur due to changes in the stratum corneum's electrical resistance, which can stimulate underlying nerves and motor neurons [86, 87]. Various drugs with different molecular weights have been administered through EP, including Fentanyl, Timolol, Orcalcein, calcitonin, and heparin, with molecular weights reaching up to 40 kDa [88]. EP involves rapidly applying a high-voltage burst, which is more effective than a prolonged low-voltage pulse. This technique offers advantages over IP, delivering macromolecules with minimal skin damage [89, 90]. The concentrations and molecular weights of the candidate drugs can influence the effectiveness of EP. Its ability to enhance molecule transport diminishes with higher concentrations and larger molecules (molecular weight > 1000). Additionally, the charge of the molecules plays a role in transportation efficacy. The electrical properties of the applied pulses and the drug concentrations are the primary factors affecting EP [91].

The electrical properties of the skin can be influenced by factors such as hydration, pH, chemical additives, electrolyte concentration, temperature, time of year, perspiration, skin conditions, thyroid activity, and emotional state. A decrease in skin resistivity, often due to increased current, voltage, or power, can enhance skin permeability. The minimum voltage required to improve skin permeability is 75 V, as lower voltages are ineffective. EP can cause side effects like skin sensations, irritation, and inflammation, with higher currents potentially leading to sharp and burning pain. Non-biocompatible components in the current-application system, such as electrodes and adhesives, can increase skin irritation risk. Despite these risks, studies have shown EP to be effective for drug delivery in various conditions if safety standards and guidelines are followed to minimize adverse effects [91–93].

### Microneedles (MN)

MNs are an innovative drug delivery method that uses arrays of microscopic needles to increase the permeability of the skin's SC. This technique offers a less invasive alternative to hypodermic needles, addressing many of their limitations and improving patient compliance. MNs create tiny pores in the skin, facilitating more effective drug absorption. In recent decades, there has been significant research and development in MN technology, highlighting its potential to transform transdermal drug delivery [94, 95]. The concept of utilizing MNs for transdermal drug delivery originated in the 1970s, but technological constraints impeded the development of these micron-scale structures. It wasn't until the late 1990s that significant progress was made, marking the beginning of practical research into MN-based TDDS [96, 97]. MNs are versatile for targeted delivery to various sites, such as the skin and eyes, but their effectiveness can vary based on the drug formulation. The ideal MN design balances size to accommodate diverse drug particles while remaining small enough to ensure patient comfort and ease of use without requiring extensive training [98]. MN functionality varies based on design (solid, hollow, coated, or dissolvable), with arrays typically comprising hundreds of MN on a substrate. MNs offer an efficient, cost-effective method for transdermal drug delivery, enhancing skin permeability to various substances while minimizing pain and tissue trauma. Despite advancements, US Food and Drug Administration (FDA) approval for MN technologies remains limited due to challenges in biomaterial selection, sterility, skin reactions, and manufacturing issues. However, MNs show promise for delivering a range of biotherapeutics, including nucleic acids, proteins, peptides, and vaccines, in precise microgram to low milligram doses [99–101].

#### *MN technology and fabrication*

MN technology has evolved significantly since its inception, with various materials and manufacturing methods being explored. Initially, silicon was the primary material used for MN fabrication, but researchers have since expanded to metals, polymers, glass, ceramics, hydrogels, and sugars. Manufacturing techniques include photolithography, etching, laser cutting, electroplating, and micro-molding [102, 103]. Each material offers unique advantages and challenges; for instance, silicon provides hardness but is fragile and expensive to process, while metals offer strength but raise safety concerns. Polymers have gained popularity due to their toughness, biocompatibility, and potential for drug encapsulation, though they may face issues with buckling during insertion [102, 104]. Dissolvable MNs have emerged as a promising category, utilizing biocompatible and biodegradable materials such as sodium hyaluronate, carboxymethylcellulose, polyvinyl alcohol, and various biodegradable polymers. These materials are selected based on their safety profiles, drug compatibility, manufacturing feasibility, and potential for large-scale production. The choice of material affects crucial factors like the dissolution rate of the skin and the preparation methods used. As MN technology advances, researchers focus on optimizing materials and manufacturing processes to enhance efficacy, safety, and scalability for diverse biomedical applications [105, 106].

#### *Types of MN*

Depending on their mechanisms and drug delivery methods, MNs are categorized into solid, hollow, coated, polymeric, and dissolvable types. Each offers unique benefits for various applications [107]:

##### Solid MN

Solid MNs create microscopic channels in the skin upon insertion, enhance skin permeability, and facilitate drug delivery through passive diffusion. This approach offers a safe method of drug administration, as the microchannels naturally close shortly after MN removal, preventing unwanted substance penetration and pathogen entry. Solid MNs effectively minimize drug leakage, thereby improving overall delivery efficiency. These MNs can be employed as a pre-treatment step to enhance skin penetration prior to applying a drug-loaded patch. By creating temporary pores in the skin, solid MNs enable drug formulations to penetrate more easily through these newly formed pathways after the MNs are removed [95, 98, 108].

##### Hollow MN

Hollow MNs function by creating conduits in the skin, allowing direct drug delivery similar to hypodermic needles. Applying pressure to flow liquid medications enables various delivery methods, including bolus, time-dependent, and slow infusion. This approach bypasses the stratum corneum, depositing drugs directly into the epidermis or dermis, which is particularly beneficial for large molecules like proteins and vaccines. Key considerations for hollow MNs include maintaining adequate mechanical strength and preventing bore clogging during drug transfer [98, 108, 109].

##### Coated MN

Coated MNs represent a specialized category of MNs characterized by a surface layer of drug-infused, typically water-soluble formulation. This innovative design allows for the direct application of therapeutic agents onto the MN structure itself. Upon insertion into the skin, the coating dissolves, delivering the drug before the MN is removed. Various techniques exist for preparing coated MNs, with studies showing they can enhance drug delivery compared to other methods. Key considerations for coated MNs include uniform coating for dose control and reproducibility, maintaining drug integrity during the coating process, compatibility between coating solution and drug formulation, drug loading capacity per MN, coating adhesion to the MN, and controlled drug release upon insertion. Common coating techniques include dip coating, roll coating, and spray coating, with dip coating often preferred due to its simplicity and ability to coat complex shapes [98, 107, 108]. The coating solution typically contains viscosity enhancers, surfactants, stabilizers, and the active drug or vaccine. Challenges include controlling the coating of specific MN sections, ensuring uniform coating across all needles in a patch, and optimizing dissolution time in the skin. The coating formulation must be water-soluble for rapid dissolution yet strong enough to adhere during insertion. Safety and compatibility with pharmaceutical manufacturing processes are also crucial considerations [98, 110–112].

##### Dissolvable MN

Dissolving MN (DM) is a polymer-based MN constructed from biocompatible, water-soluble materials such as cellulose derivatives and sugars. These MNs completely dissolve within the skin after insertion, eliminating the risk of biohazardous sharp waste [113]. Common materials used in DM include maltose, polyvinyl pyrrolidone, chondroitin sulfate, dextran, hyaluronic acid, and albumin. The dissolving nature of DM offers several advantages: it prevents tip breakage during skin insertion, eliminates concerns about insertion failure, and avoids issues associated with silicon and metal-based MNs. DMs are versatile, cost-effective, and user-friendly, making them suitable for various drug delivery applications. They don't require special training for administration, enabling patients to self-apply medications. The materials used in DMs are widely available and don't need complex preparation processes like high-temperature heating. Medications are typically formulated within the DM structure, allowing for gradual release upon insertion, similar to coated MNs. Additionally, DMs can be used as a pretreatment to enhance skin permeability [105, 114, 115].

Despite the numerous advantages of DMs, they face challenges impacting their effectiveness and reliability. One significant issue is the mechanical strength of DMs, particularly at their tips. While DMs need to be strong enough to create cavities in the SC, their tips often lack sufficient strength, negatively affecting their skin penetration reliability and consistency. This weakness can lead to incomplete skin penetration, resulting in potential drug waste and reduced efficacy. The choice of biomaterials used in DM fabrication significantly influences their skin permeability. Several techniques have been developed to fabricate and manufacture DMs, including photopolymerization, micro-molding, drawing lithography, and droplet-born air blowing. However, these fabrication methods often require harsh conditions, which can complicate the manufacturing process and raise concerns about maintaining drug integrity and standardizing dosages [105, 114–116].

Micro-molding is the most prevalent technique for fabricating polymeric MNs. This process requires a mold typically made from polydimethylsiloxane (PDMS), a commercially available silicone elastomer known for its ability to form micron-scale structures, physical stability during molding, and cost-effectiveness. Various biodegradable polymers can be used to create polymer MNs, including Poly (lactic acid) (PLA), Poly (lactic-co-glycolic acid) (PLGA), and Poly(cyanoacrylate) (PCA) and Poly(cyanoacrylate) (PCA) [113, 117]. PLGA is a biocompatible and biodegradable polymer approved by the European Medicines Agency and FDA for medical devices and TDDS use. PLGA is a linear copolymer that can be formulated in different ratios of its precursor monomers, lactic acid (LA) and glycolic acid (GA), resulting in varying degradation rates from days to months. The degradation of PLGA occurs through de-esterification, and its monomeric components are naturally eliminated from the body. A higher LA content in PLGA makes it less hydrophilic, leading to slower water absorption and degradation. Due to its versatile properties, PLGA is widely used in tissue engineering, medical imaging, and drug delivery. Polymer MNs offer several advantages over silicon-based MNs, including being less expensive, more biocompatible, and more resistant, making them suitable for mass production [117–119].

Commercial microneedle products have found diverse clinical applications across various medical fields. These include V-Go for insulin delivery in type 2 diabetes, ADAM for migraine treatment with zolmitriptan, and CorplexTM systems for Alzheimer's disease management. MicroCore PTH addresses osteoporosis with teriparatide delivery, while JewelPUMP offers another insulin delivery option for diabetes. In dermatology, products like Dermaroller®, MicroHyala®, and Dermapen® treat conditions ranging from acne to wrinkles and enhance drug absorption. Vaccine delivery benefits from technologies such as VaxMat®, Soluvia®, and IDflu®/Intanza®. Other systems like Micro-Trans®, Drugmat®, and Nanoject® offer versatile drug delivery options for various molecules and diagnostic purposes [120, 121].

### Combination of MN and IP/EP techniques for TDD

One of the most significant developments in TDDS is the combination of multiple enhancement techniques to achieve synergistic effects [34, 122]. For instance, integrating MNs with IP and EP has shown remarkable potential in improving transdermal drug delivery efficiency [123]. MNs create low-resistance aqueous pathways in the skin, which IP or EP can further exploit to increase drug flux across skin layers [36, 124]. Previous studies demonstrated that the combination of MNs, IP, and EP increased the permeation of delivery drugs compared to a single technique [125–129]. The efficacy of these combined approaches is particularly notable for delivering macromolecules, which traditionally face significant challenges in transdermal delivery due to their size. Electrical techniques also allow for better control over drug flux through modulation of the applied current, reduce the lag time for drug penetration, and offer versatility in delivering various therapeutic agents, including small molecules, macromolecules, and nanoparticles [31, 36, 124]. Recent innovations have led to developing more sophisticated and user-friendly wearable IP-driven MN patches for TDD, integrating MN's physical penetration capabilities with the electrically-driven IP drug propulsion in a single wearable device [128, 130, 131]. These integrated systems offer the potential for improved treatment outcomes, especially for chronic conditions, by enabling precise, electrically controlled drug administration through a user-friendly, wearable format. While these combined approaches show great promise, researchers also address potential limitations. Safety concerns, such as the risk of nerve stimulation due to electrical current reaching lower skin layers through MN-created channels, are being thoroughly investigated. Additionally, efforts are being made to optimize the parameters for each technique, including MN design, electrical current strength, and duration, to ensure maximum efficacy and safety for various drug types and therapeutic applications [130, 132].

## Therapeutic applications of IP/EP-assisted MN systems for enhanced drug delivery

In recent years, TDD has emerged as a promising alternative to traditional drug administration routes, offering numerous advantages such as improved patient compliance, reduced side effects, and avoidance of first-pass metabolism. As research in this field continues to evolve, electrically assisted MN systems are opening new possibilities for improving treatment outcomes across various therapeutic applications, from pain management to vaccine delivery and beyond, through improved drug administration methods [122, 124, 128, 132]. Tables 1 and 2 provide a comprehensive overview of recent studies focusing on developing and applying electrically assisted MN systems for various therapeutic approaches.

**Table 1:** Summary of studies on the application of IP-assisted MN systems for transdermal drug delivery and their therapeutic potential

Electrically assisted MN systems					
IP-assisted MN					
Delivered drug	Therapeutic application	MN and IP Characteristics	Experimental model	Results	Ref
Ovalbumin	Vaccine delivery	MN • Solid • 69 needles, 28 $\times$ 18 $\times$ 2 mm^3^ • Height (800), tip radius (20), and base diameter (400) µm IP • 0.5, 1, 1.5, and 2 mA/cm^2^ for 30 min	• Sprague–Dawley rats • BALB/c mice	• Enhanced the transdermal delivery efficiency of vaccine macromolecules	97
Rabies vaccine	Vaccine delivery	MN • Dissolving • 81 needles (3 rows $\times$ 27) • Height (257) µm, thickness (0.91) and width (7.76) mm IP • 0.5 mA/cm^2^ for 30 s followed by a recovery lapse of 30 s. for 10 min	• Skin simulant agarose gel using model drug • Wistar albino rats	• Promising for efficient, painless transcutaneous immunization	103
Glucose extraction	Diabetes	MN • Porous • Height (540), and width (300) µm IP • 0 and 0.5 mA/cm^2^ for 5 min	• H-type electrolytic cells (Porcine skin)	• Enabled passage of larger molecules through linked micropores • Potential for long-term home management of chronic diseases	110
Glucose	Diabetes	MN • Solid • 25 needles, MN hidden in touch-actuated glucose sensor with (14) mm radius and (2.7) mm thickness • Height (2.2) mm, tip radius (30), and base diameter (200) µm IP • − 0.2 to + 0.6 V, at 100 mV/s rate	• Franz diffusion cells (New Zealand rabbit skin) • Sprague–Dawley rats	• Showed high correlation with commercial glucometers in rats • Potential for painless monitoring of glucose and other interstitial fluid biomarkers	111
Insulin	Diabetes	MN • Dissolving • 138 needles, 38 $\times$ 32 $\times$ 2 mm^3^ • Height (800), tip radius (30), and base diameter (400) µm • Insulin concentration in nanovesicles: 5 IU IP • 0, 0.5 and 1 mA/cm^2^ once/ hr. for 6–12 h	• Sprague–Dawley rats	• High potential for diabetes therapy • Robust hypoglycemic effect • Negligible cytotoxicity, and good biocompatibility without skin irritation and hypersensitivity • More advanced controllability and efficiency • Potential for drug self-administration at home • More advanced controllability and efficiency	112
Insulin	Diabetes	MN • Dissolving • Needle density of 31.8 needles/ cm^2^, patch size 4.3 cm^2^ • Height (600) and base diameter (400) µm IP • 0, 0.2, and 0.5 mA/cm^2^ for 2, 5 and 10 min	• Sprague–Dawley rats	• Painless, minimally invasive • Enhanced glucose sensing and insulin delivery • Real-time monitoring and therapy with wearable design • Smart management through wireless smartphone integration	113
Insulin	Diabetes	MN • Dissolving • 225 needles, 40 $\times$ 20 $\times$ 15 mm^3^ • Height (600), tip radius (15), and base diameter (200) µm • Insulin concentration in nanovesicles: 29.3 IU/mL IP • 1, 2 and 3 mA/cm^2^ once/ hour for 12 h	• Sprague–Dawley rats	• High potential for diabetes therapy • Active and long-term glycemic regulation • Enhanced controlled insulin delivery • More advanced controllability and efficiency	114
Insulin	Diabetes	MN • Solid • 296 needles, Patch size 2 cm^2^ • Height (800), width (260) and thickness (80) µm IP • 0.2 mA/cm^2^ for 3 h	• Guinea pigs • Sprague–Dawley rats	• Increased insulin permeation • Reduced blood glucose comparable to subcutaneous injection in diabetic rats • Non-invasive peptide delivery strategy	115
Pilocarpine	Palmar sweating	MN • Micro-injection molding machine, Japan • 193 needles • Height (875) and width (190/35) μm IP • 0.5 mA/cm^2^ for 5 min	• Healthy young adults	• Increased skin permeability and sweat production on palm	116
Methotrexate	Psoriasis	MN • Dr. Pen ™ Ultima A6, USA • Height 1 mm for 10 s and microporation with a needle height of 0.5 mm for 5 s IP • 0.2 and 0.5 mA/cm^2^ for 4 h	• Franz diffusion cells (Healthy and psoriatic human skin)	• Higher Methotrexate delivery in psoriatic skin compared to healthy skin • Reduced lag time for methotrexate delivery in psoriatic skin • Faster therapeutic effect • Diseased skin shows altered drug penetration characteristics	94
Methotrexate	Psoriasis	MN • Dissolving • 28 needles • Height (500) and tip radius (3) mm IP • 0.4 mA/cm^2^ for 1 h	• Franz diffusion cells (Hairless rat skin)	• Enhanced delivery in vitro and in vivo • Synergistic enhancement in vivo compared to individual methods	117
Ropinirole hydrochloride	Parkinson's disease	MN • AdminPatch® • 187 needles • Height (600), tip width (48.64) µm IP • 0.2 mA/cm^2^ for 1 h	• Franz diffusion cells (Porcine ear skin)	• Controlled delivery of ropinirole hydrochloride for Parkinson's treatment	123
Baclofen	Multiple Sclerosis	MN • Dissolving • 81 needles (3 rows $\times$ 27) • Height (497), and base diameter (197) µm IP 0.5 mA/cm^2^ for 4 h	• Franz diffusion cells (Porcine ear skin)	• Transdermal route showed potential for baclofen administration • Offered painless, convenient delivery with improved compliance and safety • Achieved therapeutic baclofen levels for multiple sclerosis treatment	124
Diclofenac sodium	NSAID	MN • Dissolving • 100 needles, height (395) µm IP • 0.5 mA/cm^2^ for 2 h	• Franz diffusion cells (Dermatomed human skin)	• Increased absorption of diclofenac sodium • Enhanced systemic exposure of the drug	125
Ibuprofen sodium	Pain/ anti-inflammatory	MN • Dissolving • Height (610–650), and width (307–360) µm IP • 0.5 mA/cm^2^ for 15 min.—6 h	• Franz diffusion cells (Neonatal porcine skin)	• Improved delivery when combined with hydrogel-forming MN	126
Dexamethasone sodium phosphate	Hind paw oedema/ Inflammatory disorders	MN • Dissolving • 100 needles • Height (424) µm, array thickness (7.96 ± 0.13) and width of (0.95 ± 0.012) mm IP • 0.5 mA/cm^2^ for up to 300 min	• Wistar albino rats	• Reduced oedematous fluid in rat hind paws more effectively than MN alone • Reduced levels of pro-inflammatory cytokines	127
Naloxone	Opioid overdose emergency	MN • Dissolving • 225 needle • Height (600) µm IP • 0.1—0.5 mA/cm^2^ for 60 min	• Franz diffusion cells (Dermatomed human skin)	• Increased in average cumulative permeation and drug flux over a conventional dissolving MN patch • Improved desired pharmacokinetic profile for a viable naloxone delivery form through skin	128
Metformin	Obesity	MN • Dissolving • 100 needles, 10 $\times$ 10 mm^2^ • Height (800), tip radius (500), and base diameter (200) µm IP • 0.2 mA/cm^2^ for 30 min	• Obese C57BL/6 J mice	• Enhanced anti-obesity effects • Decreased body weight and fat gain • Increased energy expenditure • Reduced fat pad size • Improved energy metabolism • Enhanced browning of WAT	96, 129
Palonosetron	Chemotherapy-induced nausea and vomiting	MN • Dissolving • 225 needle, 8 × 8 mm^2^, • Height (550), base (200) and pitch (500) µm IP • 0.5 mA/cm^2^ for 30 min	• Pig skin • Sprague–Dawley rats	• MN with brief IP showed potential as a painless, self-administered, rapid-onset palonosetron delivery method • Short-duration IP enhanced delivery, achieving plasma concentrations closer to subcutaneous administration	130
Ce6(DOX)@CaCO3-NPs	Melanoma	MN • Dissolving • 100 needles, 10 × 10 array • Height (850), base (400) and base diameter (700) µm IP • 1.5 mA, 4 h	• C57BL/6 female melanoma mice	• Enabled therapeutic NPs to penetrate deep skin layers and release drugs rapidly in acidic tumor tissues • Showed superior tumor inhibition compared to the MN patch alone	131
Tetramethylpyrazine (TMP) and Borneol (BN)	Middle cerebral artery occlusion (MACO)	MN • ULTIMA-N4, Dr. pen, USA • 250–750 µm for 2 min IP • 0.4 mA/cm^2^ for 10 h	• Sprague–Dawley rats	• Synergistic enhancement of TMP delivery to brain with BN combination • Decreased infarct volumes and improved neurological scores in MCAO • Significantly increased BN permeation with combined IP and MN approach	133
Sumatriptan Succinate	Migraine headaches	MN • Dissolving • 600 needles, 0.785 cm^2^ area • Height (610–650), and width (307–360) µm IP • 100, 300, and 500 μA/cm^2^ for 6 h	• Franz diffusion cells (Mini pig skin)	• Increased drug permeation	134
Fluorescent nanoparticles	Eye diseases (Suprachoroidal space delivery)	MN • Hollow • Height (750), width (260) and thickness (80) µm IP • 0, 0.07, 0.14, or 0.7 mA/cm^2^ for 1.5, 3 or 5 min	• Rabbit eye	• Well-tolerated procedure with mild, transient effects • Lower current (0.14 mA) preferred for safety • Optimal IP time: 3 min. for posterior targeting	135

**Abbreviations:** Borneol (BN), Calcium carbonate (CaCO3) nanoparticles (NPs) loaded with chlorin e6 (Ce6) and doxorubicin (DOX) Ce6(DOX)@CaCO3-NPs, Hours (hrs.), Iontophoresis (IP), Iontophoresis-assisted microneedle (MN + IP), Microneedle (MN), Middle cerebral artery occlusion (MACO), Minutes (min.), Nanoparticles (NP), Nonsteroidal anti-inflammatory drug (NSAID), Second (sec.), Stratum corneum (SC), Tetramethylpyrazine (TMP), White adipose tissue (WAT)

**Table 2:** Summary of studies on the application of EP-assisted MN systems for transdermal drug delivery and their therapeutic potential

Electrically assisted MN systems					
**EP-assisted MN**					
**Delivered drug**	**Therapeutic application**	**MN and EP Characteristics**	**Experimental model**	**Results**	**Ref**
NP-DNA vaccine	Vaccine delivery	MN • Dissolving • 225 needles, Patch size $\approx$ 9 cm^2^ • Height (500) and base of (333) μm diameter EP • Optimal voltage 670 V/cm 6 pulses, 10 ms. per pulse with 90 ms. intervals 24–48 h	• Pig	• EP induced local inflammation, humoral and cellular immunity • EP with NPs showed highest potency • Highlights importance of testing beyond mouse models • Pig model revealed EP with DNA + NP induces strong immunogenicity	104
Nucleic acids	Dermatology and vaccination	MN • Derma roller System 600 series Germany • 10 rows of needles on the head with 60 units per row • 0.5, 1.0- and 1.5-mm long models EP • 10 electrical pulse, voltage 50, 70 or 100 V; 10 ms. pulse duration; 1 s pulse interval	• C57BL/6 J mice	• Successfully delivered gene and siRNA to mouse skin using microneedle roller-assisted electroporation • Achieved significant gene silencing with anti-SCD1 siRNA • Promising approach for in vivo delivery of various nucleic acids to skin, with potential clinical applications in dermatology and vaccination	105
Circular plasmid DNA vector	DNA vector	MN • 20 needles • Height (500), and pitch (1725) µm • Array base measured 8.62 × 6.90 mm^2^ EP • 130 V, 5 ms., 50 ms. Interval followed by 8 square wave pulses (30, 50 V, 50 ms. interval)	• C57BL/6 J mice	• Enhanced testis electroporation using microneedle-based electrodes	106
DNA and siRNA	DNA and siRNA delivery	MN • Solid • 81 needles, 20 mm^3^ • Height (190) and needles spacing (340) μm EP • 10–50 V for 20 ms. 5 pulses 2 s intervals	• HEK-293a, HeLa, MDCK and MCF-7 cells • C57BL/6 mice	• Demonstrated efficient DNA and siRNA delivery • Reduced electroporation voltage to ~ 35 V, considered safe for humans • Showed minimal tissue damage	107
Plasmid DNA	Vaccine delivery	MN • Solid • 100 needles, 200 × 200 × 500 µm^3^ • Height (400) and (200) μm at the base EP • 14–96 V, for 0.5—2 ms	• Human prostate cancer cells	• Improved intracellular delivery of proteins, DNA, and other biopharmaceuticals	108
Luciferase and Ovalbumin	Drugs and DNA delivery	MN • Hollow • 36 needles • Height (1200) and (250) μm at the base EP • HV 700 V/cm, 100 μs, LV 150 V/cm, 400 ms or 8 × 50 ms at 1 Hz • Measurements were performed 1, 30, 60 and 120 min. and 24 h. posttreatment	• Franz diffusion cells (porcine ears) • NMRI mice	• DNA electro transfer using MN for injection or as electrodes showed setup limitations • Non-activated needles disrupt electric field distribution, resulting in insufficient field for electro transfer	109
Triamcinolone acetonide	Keloid scar	MN • EPN®, Eunsung, Wonju-si, South Korea • 9 33 G-sized needles • 4,500 punctures per min • Needles inserted to a depth of 0.5 mm EP • Set at levels 2–3.5 • Procedure took approximately 5 min. and was repeated at 2–3-week intervals	• A 29-year-old woman	• Reduced the volume and height of keloid scars • Alleviated pruritus and pain • Prevented lesion recurrence	118
FITC-dextran	Dermatology	MN • Solid • 9 needles • Height (400) and (200) μm at the base EP • 50, 100, 200, and 300 V, 99 pulses with 1 ms. pulse duration	• Franz diffusion cells (Porcine skin)	• Effective combination strategy to transdermally deliver various hydrophilic macromolecules without causing structural alterations or skin damage	98
Fluorescein isothiocyanate-dextran	Dermatology	MN • Solid • 9 needles • Height (400) and (200) μm at the base EP • 50–200 V and a pulse width of 10–100 ms	• Hairless rats WBM/ILA-Ht	• MN + EP showed greater promise as a macromolecular drug delivery system for skin compared to MN alone	119
EGF	Wound healing	MN • Dissolving • 10 needles, 7 × 7 mm patch • Height (550) and base width (300) and needles spacing of (500) μm EP 20 V, 1 μA, 11 nC, 10 s	• Kunming mice	• Improved EGF pharmacodynamics • Upregulated EGF receptor expression in keratinocytes • Improved drug efficacy	120
No drug	Nerve stimulation	MN • 50, 100 needles, 8 $\times$ 8 mm^2^ • Height (410), square base (350), radius tip (30), and pitch (200) µm EP • 20 V, 1 ms	• 3D model of human skin and the blood capillaries and nerve fibers underneath	• Increased stimulation selectivity • Reduced current density due to localized electrical field • Provided more consistent current density at different tissue depths • Increasing blood flow velocity	121
No drug	Nerve stimulation	MN • Coated • 100 needles, 8 $\times$ 8 mm^2^ • Height (450) EP • 0 – 50 mA, 0.1 ms 10 – 40 V, Frequency 10 Hz to 1 kHz	• Nerve conduction velocity on hand	• Improved directivity toward target nerve • More efficient for superficial transcutaneous nerve stimulation	122
Goniothalamus macrophyllus	Cancer therapy	MN • Solid • Seirin acupuncture needles P-type 0.22 $\times$ 1.6 µm with a (2.8) mm diameter IP • 6, 9, 12 V -5 min EP • 50 V for 5 s	• Albino mice	• Elevated antioxidant markers and enzyme activities • Reduced lipid peroxidation and oxidative stress • Enhanced expression of apoptosis-related genes • Decreased anti-apoptotic and angiogenic gene expression • Improved liver and kidney function markers	92
No drug (Electrical stimulation)	Cancer Therapy	MN • Polymeric • 100 needles • Height (700) and needles spacing of (800) μm EP • 1 V, 100 kHz sine wave, for 1 h	• C57BL/6 and ICR mice	• Induced necrotic cell death in cancer cells • Released of damage-associated molecular patterns, activating immune response • Systemic anti-tumor effects • Inhibited metastasis, and tumor growth	132

**Abbreviations:** Electroporation (EP), Electroporation-assisted microneedle (MN + EP), Electroporation microneedling device (EPN), Epidermal growth factor (EGF), FITC-labeled bovine serum albumin (BSA), Fluorescein isothiocyanate (FITC), hours (hrs.), Iontophoresis (IP), Iontophoresis and electroporation–assisted microneedle (MN + IP + EP), microneedle (MN), Middle cerebral artery occlusion (MACO), Minutes (min.), Nanoparticles (NP), plasmid DNA encoding green fluorescent protein (GFP), Second (sec.), Tetramethylpyrazine (TMP)

In studies conducted by Zheng et al. [128], Arshad et al. [133], Bernelin-Cottet et al. [134], Huang D et al. [135], Houlihan R et al. [136], Wei et al. [137], Choi et al. [138], and Daugimont L et al. [139], IP or EP-assisted MN approaches were used for vaccine, DNA and/or siRNA delivery. The studies demonstrated enhanced vaccine delivery using MN with uniform projections, high swelling capacity, and durability. These findings suggest IP/EP-assisted MN approaches offer significant advantages for efficient, painless transcutaneous immunization, potentially improving vaccine effectiveness and patient compliance.

The combined application of MN and IP has been investigated in several recent studies, revealing significant potential for diabetes management [140–145]. These methods showed high potential for diabetes therapy, offering robust hypoglycemic effects, enhanced glucose sensing, and controlled insulin delivery. The systems exhibited good biocompatibility, negligible cytotoxicity, and minimal skin irritation. Wearable designs integrated with smartphones enable real-time monitoring and smart management, suggesting the potential for long-term home management of chronic diseases [141, 143]. Combining MNs with charged nanovesicles and IP further increased insulin permeation, achieving blood glucose reduction comparable to subcutaneous injections in diabetic rats [142, 144, 145].

The study by Amano T et al. [146] demonstrated that MN application effectively disrupted the skin barrier function on palmar skin. This pretreatment significantly enhanced iontophoretic pilocarpine-induced palmar sweating. Interestingly, similar MN pretreatment on forearm skin did not affect pilocarpine-induced sweating. Notably, the observed palmar sweating increased without a corresponding increase in pilocarpine delivery. These findings suggest that MN pretreatment specifically enhances the sweat response in palmar skin, potentially through mechanisms independent of increased drug penetration. IP/EP-assisted MN approaches have shown significant promise in enhancing TDD for various skin conditions. Studies by Vora D et al. [125] and Vemulapalli V et al. [147] demonstrated higher methotrexate delivery in psoriatic skin than healthy skin, with IP reducing lag time for drug delivery. The combination of MN and electrical methods exhibited synergistic enhancement in vivo, effectively delivering hydrophilic macromolecules without causing skin damage. This approach also proved beneficial in treating keloid scars, reducing scar volume, alleviating symptoms, and preventing recurrence [148]. Additionally, the technique showed potential for in vivo delivery of nucleic acids to skin [135], with applications in dermatology [129, 149]. Yang et al. [150] developed a microneedle-based self-powered transcutaneous electrical stimulation system (mn-STESS) to enhance epidermal growth factor delivery in wound healing. The system improved drug penetration and generated electrical stimulation as an adjuvant to overcome pharmacodynamic challenges.

Studies by Soltanzadeh et al. [151, 152] demonstrated that MN electrodes for transcutaneous electrical nerve stimulation offer significant advantages over conventional surface electrodes, including lower contact resistance, higher selectivity, improved deep-layer current density, and more consistent stimulation across various tissue depths. These findings suggest MN electrodes can enhance the efficiency and precision of nerve stimulation in clinical applications. Singh et al. [153] study found that IP-assisted MN delivery significantly enhanced transdermal ropinirole hydrochloride delivery. This method offers precise control for dose titration in Parkinson’s disease therapy. Furthermore, Junaid MSA et al. [154] have demonstrated the application of IP-assisted MN and the potential for baclofen delivery in multiple sclerosis treatment. This transdermal route offers a painless and convenient delivery method, improving patient compliance and safety while achieving therapeutic baclofen levels. Similarly, this method has significantly enhanced the transdermal delivery and systemic absorption of NSAIDs, including diclofenac sodium [155] and ibuprofen sodium [156]. This approach, particularly when used with hydrogel-forming MN, improves the delivery of these pain and anti-inflammatory drugs, potentially offering more effective treatment options. Another study by Arshad, M.S. et al. [157] showed that combining MN-loaded dexamethasone sodium phosphate with IP significantly reduced oedematous fluid in rat hind paws and lowered pro-inflammatory cytokine levels more effectively than MN alone.

Tijani et al. [158] developed an IP-assisted dissolving MN that significantly improved transdermal naloxone delivery for opioid overdose emergencies. This optimized design increased drug permeation and flux, potentially offering a viable alternative to current naloxone administration methods. In studies by Abbasi et al., the combined use of MN and IP for metformin delivery enhanced anti-obesity effects, leading to decreased body weight and fat gain, increased energy expenditure, reduced fat pad size, improved energy metabolism, and enhanced browning of WAT. They have indicated that combining IP and MN could significantly enhance local drug delivery to WAT compared to the liver, reducing the therapeutic dose to 3 mg/kg BW/day [127, 159].

IP and/or EP-assisted MN have been utilized in cancer therapy to improve the delivery of palonosetron [160] and goniothalamus macrophyllus [36], effectively managing side effects like nausea and vomiting. Combining MNs with brief IP offered a painless, self-administered method for rapid palonosetron delivery, achieving plasma concentrations similar to subcutaneous administration. This approach enhances antioxidant markers, reduces oxidative stress, promotes apoptosis-related gene expression, decreases anti-apoptotic and angiogenic gene expression, and improves liver and kidney function markers. In a study by Wang et al., [161] a wearable self-powered MN for melanoma treatment enabled therapeutic nanoparticles to penetrate deep skin layers and release drugs rapidly in acidic tumor tissues, showing superior tumor inhibition compared to the MN patch alone. A flexible MN-array-integrated interdigital electrode (FMIE) was developed by Pan et al. [162] for cancer treatment. The FMIE induced cancer cell death, activated immune responses, and inhibited tumor growth and metastasis, offering a promising drug-free therapy approach.

MN-mediated transdermal delivery of tetramethylpyrazine to the brain, enhanced by borneol and IP, effectively prevents middle cerebral artery occlusion. This combination significantly improves tetramethylpyrazine delivery to the brain, reduces infarct volumes, and enhances neurological scores while increasing borneol permeation through the integrated IP and MN approaches [163]. IP-assisted MN has also been tested for delivering sumatriptan succinate for migraine headaches, demonstrating increased drug permeation [164]. Additionally, this method has been evaluated for delivering fluorescent nanoparticles to treat eye diseases via the suprachoroidal space. The procedure is well-tolerated, with mild, transient effects, and a lower current is preferred for safety [165].

## Challenges and future perspectives

IP and/or EP-assisted MN presents significant challenges and promising future perspectives in TDD. One of the primary challenges is ensuring safety, as electrical currents can cause skin irritation, burns, or tissue damage if not properly controlled. Long-term effects of repeated electrical stimulation also remain a concern. Development challenges include optimizing MN design for effective drug delivery and patient comfort, integrating electrical components into a wearable, user-friendly device, and ensuring consistent and controlled drug delivery across different skin types. Scaling up manufacturing processes for commercial production adds another layer of complexity [29, 166, 167].

Clinical applications of IP and/or EP-assisted MN are currently limited to specific drug types, mainly small molecules and some peptides, which may not be suitable for all patient populations, particularly infants and the elderly [168, 169]. This limitation underscores the importance of thorough patient education and adherence to treatment protocols. Technological challenges include optimizing battery life in wearable devices, ensuring uniform drug distribution, and adjusting electrical parameters to achieve effective delivery without causing discomfort [101, 170, 171]. These limitations highlight the need for ongoing research to expand the range of compatible drugs and improve device performance. Future advancements may focus on enhancing energy efficiency, developing sophisticated drug delivery mechanisms, and creating adaptable systems for a broader patient demographic [172, 173].

The future of electrically assisted MN technologies in TDD is promising, with potential advancements in safety, technology, and clinical applications [101]. Anticipated improvements include smart systems for real-time skin monitoring, biocompatible materials, and personalized treatment protocols. Technological progress may lead to integrated sensing technologies, miniaturized components, and dissolvable MN with built-in electrodes. Clinical applications could expand to larger molecule delivery, targeted delivery to specific skin layers, and combination therapies. Future research may focus on optimizing drug formulations for electro-assisted delivery, including nanocarriers for improved stability and penetration. Personalized medicine approaches and integration with wearable health monitoring devices could enable tailored treatments. Non-invasive alternatives and combination methods may further expand the field. Establishing regulatory guidelines and standardized testing methods will be crucial for widespread adoption [101, 170, 174].

## Conclusion

Electrically assisted MN technologies, such as IP and EP, represent a significant advancement in TDD. These technologies bring both challenges and opportunities. Safety concerns, design optimization, and technological limitations currently preventing widespread adoption. However, advancements in smart systems, biocompatible materials, and personalized treatment plans can potentially improve patient outcomes. These technologies may expand to include the delivery of larger molecules and targeted therapies. Innovations in drug formulations and integration with wearable health devices could enable more personalized treatments. Establishing regulatory guidelines and standardized testing is essential for ensuring the safety and effectiveness of these systems as the field advances. Ongoing research and development in electrically assisted MN technologies can revolutionize TDD, providing enhanced treatment options in various therapeutic areas, such as pain management and vaccine delivery, while improving patient compliance and satisfaction overall.

## Data Availability

This review article is based on the analysis and synthesis of previously published studies. All data and materials referenced in this review are available in the cited publications. No new data were generated or analyzed in this study.

## Abbreviations

dWAT: Dermal White Adipose Tissue
EP: Electroporation
EP-MN: Electroporation-Mediated Microneedle
FMIE: Flexible microneedle-array-integrated interdigital electrode
IP: Iontophoresis
IP + EP-MN: Iontophoresis and Electroporation-Mediated Microneedle
IP-MN: Iontophoresis Mediated Microneedle
LA: Lactic Acid
mn-STESS: Microneedle-based self-powered transcutaneous electrical stimulation system
NSAIDs: Nonsteroidal Anti-Inflammatory Drugs
PLA: Poly (Lactic Acid)
PLGA: Poly (Lactic-Co-Glycolic Acid)
PCA: Poly (Cyanoacrylate)
SC: Stratum Corneum
sWAT: Subcutaneous White Adipose Tissue
TDD: Transdermal Drug Delivery
TDDS: Transdermal Drug Delivery Systems
WAT: White Adipose Tissue

## Data availability

No new data were created or analyzed in this study. Data sharing is not applicable to this article.
